# Impact of iron deposit on the accuracy of quantifying liver fat fraction using multi‐material decomposition algorithm in dual‐energy spectral computed tomography

**DOI:** 10.1002/acm2.13368

**Published:** 2021-07-20

**Authors:** Dandan Du, Xingwang Wu, Jinchuan Wang, Hua Chen, Jian Song, Bin Liu

**Affiliations:** ^1^ Department of Radiology First Affiliated Hospital of Anhui Medical University Hefei Anhui China

**Keywords:** dual‐energy CT, fat quantification, IDEAL‐IQ, iron deposit, MRI, multi‐material decomposition

## Abstract

**Objectives:**

To investigate the accuracy of using multi‐material decomposition (MMD) algorithm in dual‐energy spectral computed tomography (CT) for quantifying fat fraction (FF) in the presence of iron.

**Materials:**

Nine tubes with various proportions of fat and iron were prepared. FF were divided into three levels (10%, 20%, and 30%), recorded as references (FF_ref_). Iron concentrations (in mg/100 g) were divided into three ranges (25.25–25.97, 50.38–51.55 and 75.57–77.72). The nine‐tube phantom underwent dual‐energy CT and MR. CT attenuation was measured and FF were determined using MMD in CT (FF_CT_) and Iterative Decomposition of water and fat with Echo Asymmetry and Least squares estimation (IDEAL‐IQ) in MR (FF_MR_) for each tube. Statistical analyses used were: Spearman rank correlation for correlations between FF_ref_ and CT attenuation, FF_CT_, and FF_MR_; one‐way ANOVA, and one‐sample *t*‐test for the differences between FF_CT_ and FF_ref_ and between FF_MR_ and FF_ref_. A multivariate linear regression model was established to analyze the differences between the corresponding values with different iron concentrations under the same FF_ref_.

**Results:**

Fat fraction on CT (FFCT) and FF_MR_ were positively correlated with FF_ref_ (all *p* < 0.001), while the CT attenuation was negatively correlated with FF_ref_ in the three iron concentration ranges. For a given FF_ref_, FF_CT_ decreased and FF_MR_ increased as the iron concentration increased. The mean difference between FF_CT_ and FF_ref_ over the nine tube measurements was 0.25 ± 2.45%, 5.7% lower the 5.98 ± 3.33% value between FF_MR_ and FF_ref_ (*F* = 310.017, *p* < 0.01).

**Conclusion:**

The phantom results indicate that MMD in dual‐energy CT can directly quantify volumetric FF and is less affected by iron concentration than MR IDEAL‐IQ method.

Nonalcoholic fatty liver disease (NAFLD) is becoming the most common chronic liver pathology both in Chinese and Western countries. About 30% of the population is at risk, fatty liver is often associated with iron metabolism disorders,^1^ of which up to 30% of patients have hyperseroferritinemia and liver iron deposition, which is called dysmetabolic iron overload syndrome.^2^, ^3^, ^4^ Therefore, it is important to evaluate the severity of hepatic steatosis with iron overload. Liver biopsy has remained the “gold standard” mainly for assessment of liver fat content. However the liver biopsy sample only accounts for 0.002% of the entire liver volume, so there is sampling errors in this method, and liver biopsy is an invasive surgery with complication rates of about 0.6%−18%.^5^, ^6^

Noninvasive quantitative methods for expressing liver fat concentration, therefore, have attracted more and more clinical attention, include those related to ultrasound (US), magnetic resonance (MR), and computed tomography (CT). US is widely used for the assessment of hepatic steatosis; however, it is relatively insensitive and nonspecific for the detection of individuals with mild steatosis.^7^ To our knowledge, there is no established quantitative US for assessing fatty infiltration of the liver. At present, there are two kinds of MR sequences used for fat quantification: magnetic resonance spectroscopy (MRS) and Iterative Decomposition of water and fat with Echo Asymmetry and Least squares estimation (IDEAL‐IQ), which have been verified in fat–water phantoms and animal models,^8^, ^9^, ^10^, ^11^, ^12^ However, iron deposition in the liver can change the uniformity of magnetic field, resulting in inaccurate measurement.

In the past, attenuation values derived from the single‐energy CT is used to estimate density and is semi‐quantitative for measuring liver fat content. However, due to the scanning parameters and the presence of iron, glycogen, drugs, or other substances, the measured fat content varies from patient to patient.^13^ In addition, the beam‐hardening artifact has been pointed out as a cause for the attenuation value to drift, which further affects the semi‐quantitative results. And researchers believe that the CT attenuation values are not sensitive to mild hepatic steatosis.^14^ On the other hand, the monochromatic images in dual‐energy spectral CT can correct the beam‐hardening effect, making the attenuation value more accurate and less affected by the shapes of the objects and the measurement locations in the objects than the polychromatic images.^15^, ^16^ Multi‐material decomposition (MMD) is a new technology in dual‐energy spectral CT imaging that can distinguish two or more different substances at the same time,^17^, ^18^ and quantitatively and intuitively evaluate liver fat content in volume to generate volume fat fraction (FF).

The purpose of our study was to validate the accuracy of using MMD algorithm in dual‐energy spectral CT for measuring FF using a fatty liver phantom with different FFs and iron concentrations, and to study the effect of iron on the measured FF accuracy, and to compare them with MR IDEAL‐IQ measurements against the ground truth.

## 1 MATERIALS

### 1.1 Liver phantom preparation

Fresh swine liver was purchased from market and MR IDEAL‐IQ scan was performed to confirm that the fat concentration in the liver was less than 2%. The blood vessels and bile duct tissue were first removed from the swine liver before it was grinded and filtered. The liver was then deaerated with a vacuum deaerator for 30 min at room temperature. Nine mixtures that contained different proportions (Table 1) of the swine liver, pure lard, iron (Iron sucrose Injection, Nanjing Hengsheng Pharmaceutical Co., Ltd; 100 mg of iron per 5 ml ampule), and distilled water were prepared and again deaerated until air bubbles were undetectable for 30 min. These mixtures were put in nine tubes and placed into a polypropylene phantom of 20 cm diameter (QSP‐1; Fuyo Corporation). Based on the literatures, the nonalcoholic liver steatosis is graded in the following way: grade 0, 0%–6.4% (normal); grade 1, 6.5%–17.4% (mild); grade 2, 17.5%–22.1% (medium); and grade 3, 22.2% or higher (severe).^19^, ^20^ Therefore, in our study the nine tubes were prepared to represent the three diseased levels at 10%, 20% and 30% and recorded as reference levels for the fat fraction (FF_ref_). In each of the three tubes with same FF level, 0.5, 1.0, and 1.5 ml of iron sucrose were added by injection. The nine mixtures were weighed, and the iron concentration was calculated to be at roughly three levels (25.25–25.97, 50.38–51.81, and 75.57–77.72 mg/100 g). Iron content was reported to be less than 40 mg per 100 g of liver (wet weight) in healthy individuals.^21^ In our experiment, the molecular formula of the soluble sucrose iron added was C6H8FeO8 (Fe^+2^),^22^ simulating the relatively normal and high level of iron content in the liver. See Table 1.

**TABLE 1:** Material composition of the tubes

Tube no.	Swine liver (ml)	Lard (ml)	Iron^a^ (ml)	Distilled water (ml)	FF_ref_ (%)	Iron content (mg/100 g)
1	30	4	0.5	5.5	10	25.25
2	30	4	1.0	5.0	10	50.38
3	30	4	1.5	4.5	10	75.57
4	26	8	0.5	5.5	20	25.58
5	26	8	1.0	5.0	20	51.81
6	26	8	1.5	4.5	20	78.74
7	22	12	0.5	5.5	30	25.97
8	22	12	1.0	5.0	30	51.55
9	22	12	1.5	4.5	30	77.72

Abbreviation: FF, fat fraction.

^a^ Iron sucrose injection (20 mg of iron per milliliter).

### 1.2 CT acquisition and data analysis

**FIGURE 1:**
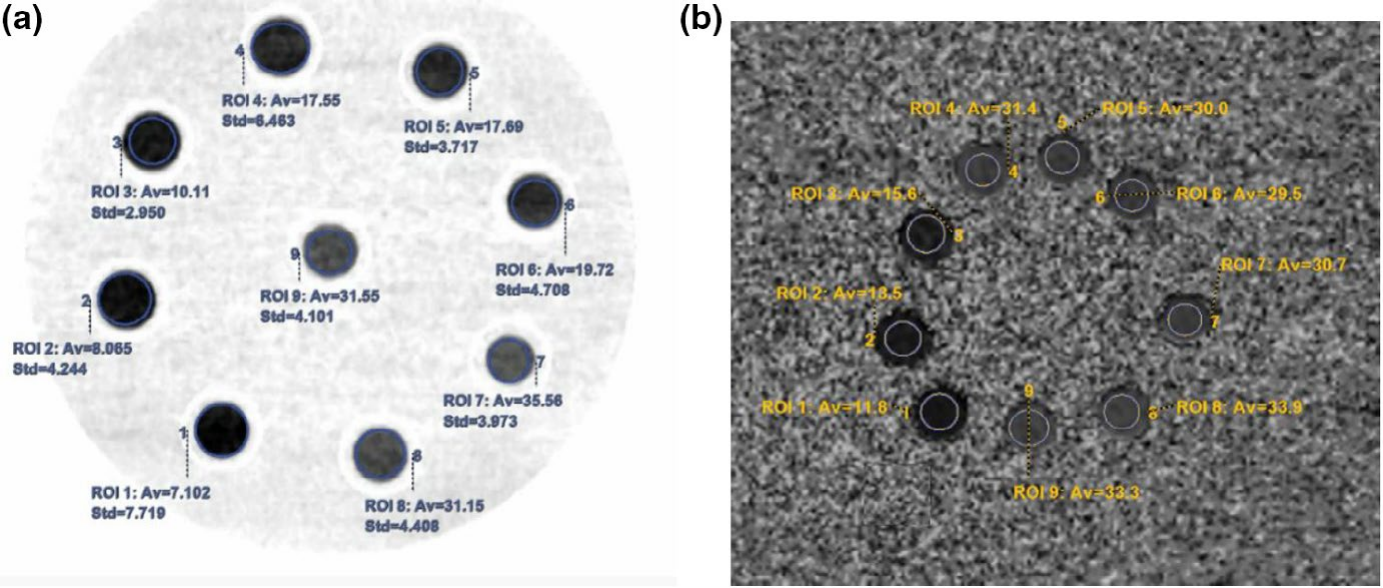
The exact values in nine ROI tubes by CT and MR fat fraction images. (a) Fat fraction image from MMD; (b) Fat fraction image from IDEAL‐IQ. The numbers correspond to the nine tubes shown in Tables 1 and 2. IDEAL‐IQ, Iterative Decomposition of water and fat with Echo Asymmetry and Least squares estimation; MMD, multi‐material decomposition; MR, magnetic resonance; ROI, region of interest

The QSP‐1 phantom that contained the nine tubes was scanned on a 256‐section multidetector CT scanner (Revolution CT; GE Healthcare) using the dual‐energy CT imaging mode. The scan parameters were as follows: tube voltage 80/140 kVp instantaneous switching; Tube current: 200 mA; Noise index: 6; gantry rotation speed: 0.5 r/s. Scan was repeated three times and the CT dose index value for each scan was 2.99 mGy. Images were reconstructed at layer thickness/spacing of 5.0 mm/1.25 mm using a new version adaptive statistical iterative reconstruction (ASIR‐V) with 40% strength. The 120 kVp‐like CT images were generated to measure CT attenuation in Hounsfield units (HU). The images were transmitted to an advanced workstation (AW Volumeshare 7; GE Healthcare) which was equipped with a currently not‐yet‐commercially available MMD software (GE Healthcare) to measure the FF_CT_ (fat fraction on CT MMD image; Figure 1a). The MMD image is obtained from two sets of measurements associated with low and high energies from dual‐energy spectral CT measurements. This method assumes three basis materials at the most within each pixel, and the material types alter among the pixels. It uses volume conservation to obtain three materials for reconstruction and decomposition to obtain live fat images for quantifying fat fraction.

Immediately after the CT scan, MR IDEAL‐IQ imaging were performed on the nine tubes placed on the plate by using a 3.0 T MR clinical imager (Discovery 750 W; GE Healthcare) with a 24‐channel head–neck combined coil. The IDEAL‐IQ sequence had the following acquisition parameters: scan plane: Axial, TR: 7.4 ms, TE:1–5.3 ms, number of echoes 3, display field‐of‐view: 40 cm, matrix size: 160 mm×160 mm; flip angle 4°; Slice Thickness: 3 mm; space between slices 0 mm. Scan was repeated three times. The MR fat fraction (FF_MR_) maps were generated by post‐processing software provided by the manufacturer (Figure 1b).

Two abdominal radiologists (more than 5 years of abdominal CT and MRI diagnosis experience) performed the measurement independently. CT and MR measurements were performed by placing a region of interest (ROI, 140 mm^2^, 3 mm deviation) in the center of each tube over three consecutive image slices. The mean and SD of the measured values of each tube were calculated by averaging measurements over the three image slices and by the two radiologists (see Table 2).

**TABLE 2:** Measurement values of fatty liver phantoms with different iron concentrations (mean and SD)

Tube no.	FF_ref_ (%)	Iron (mg/100 g)	CT attenuation (HU)	FF_CT_ (%)	FF_MR_ (%)
1	10	25.25	40.78 ± 3.13	8.50 ± 0.98	12.77 ± 0.78
2	10	50.38	43.98 ± 1.87	8.27 ± 1.62	13.39 ± 0.42
3	10	75.57	46.24 ± 1.54	7.61 ± 1.44	15.67 ± 0.57
4	20	25.58	22.90 ± 2.63	20.45 ± 1.89	29.72 ± 2.18
5	20	51.81	25.09 ± 2.15	20.39 ± 1.81	29.97 ± 1.21
6	20	78.74	28.06 ± 2.07	19.58 ± 2.19	30.60 ± 1.67
7	30	25.97	3.28 ± 2.07	33.03 ± 1.76	32.90 ± 1.20
8	30	51.55	6.02 ± 2.41	32.82 ± 1.63	33.49 ± 0.56
9	30	77.72	11.93 ± 1.72	31.63 ± 1.81	35.29 ± 1.96

### 1.3 Statistical analysis

The original data were verified and analyzed with SPSS 17.0 statistical software. Spearman rank correlation was used to analyze the correlation between FF_ref_ and corresponding CT measurements (dual‐energy CT FF_CT_ and CT value) and MR measurements (IDEAL‐IQ FF_MR_). One‐way ANOVA and one‐sample *t*‐test were used to compare the measurement errors from the gold standard (FF_ref_) between the dual‐energy CT FF_CT_ and IDEAL‐IQ FF_MR_; A multivariate linear regression model was establish to analyze the differences between the corresponding values with different iron concentrations under the same FF; setting test Level *α* = 0.05.

## 2 RESULTS

**FIGURE 2:**
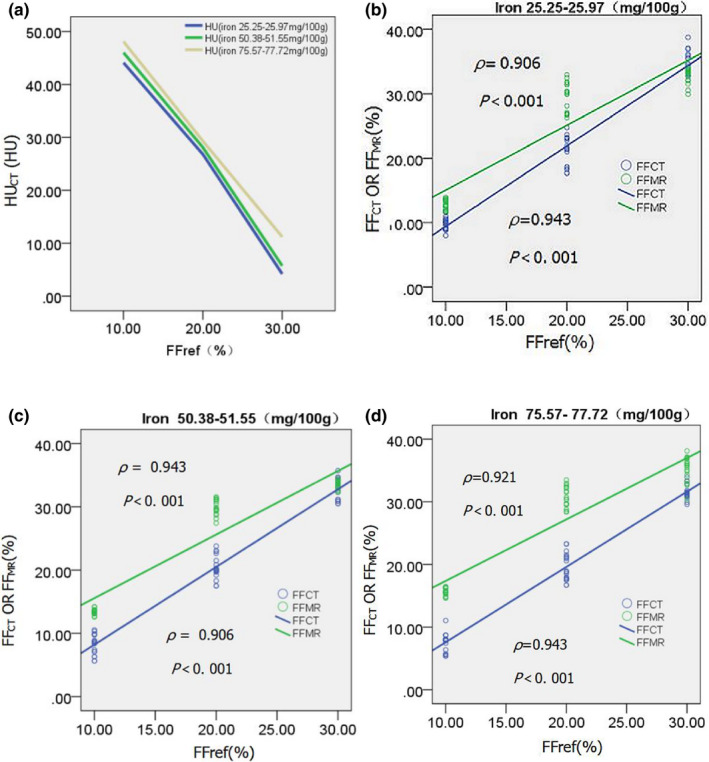
(a) Line charts show the corresponding changes on CT attenuation (HU) with the increase of FF_ref_ by using a 120 kVp‐like image with three levels of iron concentration. (b–d) Scatterplots with superimposed regression lines of FF_ref_ versus CT and MR measurements for the phantom with three levels of iron concentration. The correlation coefficients of Spearman and associated *p* values are shown. FF, fat fraction; HU, Hounsfield units; MR, magnetic resonance

**FIGURE 3:**
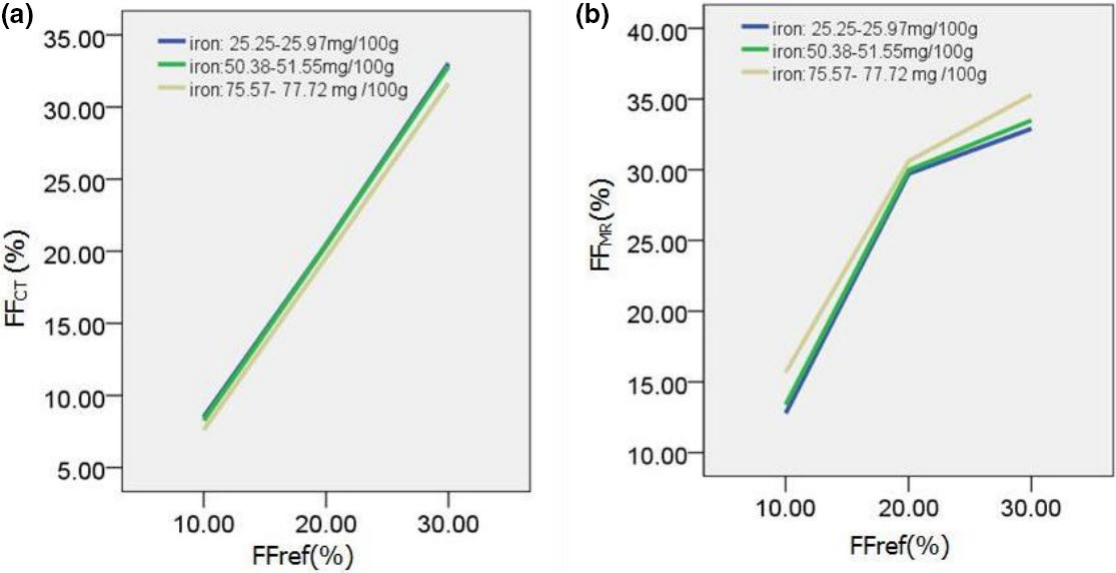
Line charts of the effect of different iron concentrations on FF_CT_ (a) and FF_MR_ (b) measurements. FF, fat fraction; MR, magnetic resonance

Figure 2a shows the change of CT attenuation value (on a 120 kVp‐like image) as a function of FF_ref_, affected by the three levels of iron concentration (25.25–25.97, 50.38–51.55, and 75.57–77.72 mg/100 g). The line charts showed that CT attenuation value was negatively correlated with FF_ref_. Figure 2b–d show the scatterplots of the measured values of FF_CT_ on MMD and FF_MR_ on MR DEAL‐IQ as function of FF_ref_, affected by the three levels of iron concentration. The three groups all showed good correlations: FF_ref_ positively correlated with dual‐energy CT FF_CT_ (*ρ* = 0.943, *ρ* = 0.906 and *ρ* = 0.943) and MR IDEAL‐IQ FF_MR_ (*ρ* = 0.906, *ρ* = 0.943 and *ρ* = 0.921) for the three iron concentration levels, respectively (all *p* < 0.001). Figure 3 shows the changes of FF_CT_ and FF_MR_ measurement values under different iron concentrations indicated that FF_CT_ decreased and FF_MR_ increased with the increase of iron concentration under the same FF_ref_ value.

The mean difference over the nine tube measurements between FF_CT_ and FF_ref_ (named group A) was 0.25 ± 2.45%, and the difference between the FF_MR_ and FF_ref_ (named group B) was 5.98 ± 3.33%. A one‐way ANOVA was used to statistically evaluate the differences, and the results suggested that the difference between group A and group B was statistically significant (*F* = 310.017, *p* < 0.01). A one‐sample *t*‐test was performed on the overall sample: there was no statistical difference between group A and FF_ref_ with a mean of 0 (*t* = 1.313, *p* = 0.191), while group B was statistically different from FF_ref_ with a mean of 0 (*t* = 22.833, *p* < 0.01), indicating that overall the measured value from CT MMD was more consistent with FF_ref_, and the measured value from MR IDEAL‐IQ had systematic positive bias compare with FF_ref_.

Using the FF measurement results from CT MMD and MR IDEAL‐IQ scan methods as the dependent variable, the actual FF, iron solution concentration, and scan method were used to fit the multiple linear regression model for the independent variables. The specific variable assignments are given in Table 3. An entrance tolerance of 0.05, and an exit tolerance of 0.10. The regression analysis indicated that the regression equation had a good fitting degree (*F* = 1251.640, *p* < 0.01) and multiple regression analysis with a determination coefficient of *R* ^2^ = 0.922. The results are given in Table 3. According to the nonstandardized regression coefficients of the measurement methods, under the condition that other variables were fixed, the mean value of the MR IDEAL‐IQ FF measurement was 5.7% higher than the mean value of the CT MMD measurement (*p* < 0.01). Specifically, there was underestimation of the FF by MMD measurement in dual‐energy CT (FF_CT_) for the low FF setting, and overestimation by MRIDEAL‐IQ (FF_MR_) at all three FF settings, which further illustrated the statistical difference between the FF measurement values of the two scanning methods (*p* < 0.01). The results also showed that there was no statistical difference in the effect of iron content on the measurement results of the two groups. (*p* = 0.186).

**TABLE 3:** Multiple stepwise linear regression analysis results

Variable	Nonstandardized coefficient		Normalization coefficient (*β*)	*t*	*p*
	*B*	Standard error			
Constant term	−8.107	0.726	—	−11.163	<0.01
FF_ref_	1.108	0.019	0.916	58.440	<0.001
Iron solution content	0.502	0.379	0.021	1.324	0.186
Measurement method	5.700	0.310	0.288	18.384	<0.01

## 3 DISCUSSION

In principle, dual‐energy CT can only accurately reconstruct two materials with different attenuation coefficients when performing scanning measurements under high and low‐energy fast switching conditions.^16^, ^17^ In contrast‐enhanced scans, liver fat quantification requires the differentiation of four materials: liver tissue, blood, fat, and contrast agent. This results in limited clinical use of current material decomposition techniques. MMD algorithm is a flexible model‐based method developed for dual‐energy CT, which can expand the core material discrimination ability of dual‐energy CT and discriminate among two or more different materials at the same time. MMD algorithm has two main clinical applications for contrast‐free dual‐energy CT data or contrast‐enhanced dual‐energy CT data, the first is virtual un‐enhancement, the second is liver‐fat quantification.^23^ The algorithm used in our phantom study was for LFQ, which is performed with fat and healthy liver tissue in the material basis, and uses volume conservation to obtain three materials for reconstruction and decomposition for fat quantification, and obtains live fat images for quantifying FF.^23^ The second generation of dual‐energy spectral CT (GE revolution CT) was used in our phantom study, which is superior to the previous generation (GE discovery CT). It adopts more advanced ASIR‐V, and further optimizes the performance of multi‐material qualification, quantification, and noise reduction.

In our study, the mixture of swine liver and fat was used to simulate the state of human fatty liver. In preparing the liver phantom, the blood vessels and bile duct tissue were first removed from the swine liver before it was grinded and filtered, and then deaerated with a vacuum deaerator. As swine liver tissue contains more bile duct and blood vessel components compared with human liver tissue, the CT attenuation of swine liver is higher than that of human liver tissue and the removal of bile duct and blood vessel would make the CT attenuation in the phantom closer to that of human liver tissue. It is worth noting that gas will be mixed in the process of preparing the fatty liver phantoms, which will also affect the measurement values. Therefore, these preparations are the key steps to prepare a qualified fatty liver phantom, so as to make the measurement results more accurate.

At present, there are not many studies on the effect of liver iron deposit on the quantitative measurement of fat in nonalcoholic fatty liver. Eskreis‐Winkler et al. verified the accuracy of IDEAL‐IQ in 10 cancer patients with both elevated liver fat and elevated liver iron, and found that the measurement value of IDEAL‐IQ fat content was more accurate than that of IOP (T1 in and out of phase).^24^ Furthermore, Catherine et al. verified the accuracy of MRS on fat‐fraction quantitative in the mice model of steatosis and iron overload, and found that there was an excellent correlation between MRS fat‐fraction and group based fat quantitative.^25^

Our phantom results on the dual‐energy CT MMD measurement value FF_CT_, 120kvp‐like CT measurement value (HU), and MRI IDEAL‐IQ measurement value FF_MR_ demonstrated a strong correlation with FF_ref_ in the presence of different iron concentrations. However, for CT examination, the advantage of the MMD method over CT attenuation is the ability of the former to intuitively measure fat content in units of volume percentage. Our study results validated that the agreement in the measurement value of dual‐energy CT MMD fat content was better than that of MRI IDEAL‐IQ in the presence of iron. One previous phantom study by Tomoko Hyodo et al. assessed the influence of iron on fat measurements determined with Dual‐energy CT (DECT) and MRS by using mixtures with iron concentration at three levels (0, 48.1–55.9, and 92.6–103.0 mg/100 g), and found that iron concentration had less influence on the measurement of FF by CT MMD, but led to the underestimation and overestimation of fat content measurements with MRS. Our phantom study also showed that the average value of the FF_MR_ was higher than FF_CT_, specifically in the presence of iron, FF_CT_ was underestimated in low fat concentrations and FF_MR_ was overestimated in low, medium, and high fat concentrations. Therefore, the MMD algorithm may produce fewer errors in patients with fatty liver and high iron content. For example, patients with chronic liver disease with excessive iron may get more accurate results with dual‐energy CT. The range of iron concentration in our study was 25.25–77.72 mg/100 g, which cannot represent the concentration range of iron deposition for all diseases, such as genetic hemochromatosis (more than 100 mg iron per 100 g liver [wet weight]), etc, which needs further verification.

An unexpected result was that under the same FF_ref_ condition, with the increasing amount of iron the FF_CT_ measurement value gradually decreased, and the FF_MR_ measurement value gradually increased, indicating that the changes in iron concentration had a decreasing and increasing trend on CT and MR measurements. The results of multiple linear regression model analysis showed that iron concentration had similar impact of the measurement errors for CT MMD and MR IDEAL ‐IQ measurements regardless of the amount of fat content (*p* = 0.186). This indicated that both the MMD algorithm and IDEAL‐IQ had good correction for the influence of iron.

There are a number of limitations in this study: (1) We did not make precise chemical extraction of the fat concentration and iron concentration in the fatty liver model; (2) The model did not cover the cases of clinically high‐concentration liver iron; (3) A single DECT protocol was used in our study and the effect of altering CT acquisition and reconstruction parameters was not studied; (4) In order to avoid the interference of the phantom on the magnetic field stability during MR scanning, the tubes were placed in the air which may have some impact on the measurement results.

In summary, our phantom results demonstrate a strong correlation and consistency between the measurement results of MMD and the actual FF, and indicate that DECT based MMD method is promising for noninvasive quantification of hepatic steatosis with lower susceptibility to iron, which is of great importance for the diagnosis, characterization, and treatment of fatty liver disease, and has broad prospects for clinical application.

## AUTHORS' CONTRIBUTIONS

D.D., X.W., J.W. and H.C. purchased experimental materials, prepared liver phantom and measured experimental data, and D.D. drafted the manuscript. J.S. scanned the phantom. B.L. participated in its design and coordination.

## Data Availability

The datasets generated during and analyzed during the current study are available from the corresponding author on reasonable request.
